# The apparent permeabilities of Caco-2 cells to marketed drugs: magnitude, and independence from both biophysical properties and endogenite similarities

**DOI:** 10.7717/peerj.1405

**Published:** 2015-11-17

**Authors:** Steve O’Hagan, Douglas B. Kell

**Affiliations:** School of Chemistry & The Manchester Institute of Biotechnology and Centre for Synthetic Biology of Fine and Speciality Chemicals (SYNBIOCHEM), The University of Manchester, Manchester, Lancs, United Kingdom

**Keywords:** Caco-2 cells, Facilitated diffusion/transport, Permeability, Oral absorption, Transcellular transport, Mathematical models, Transporter-mediated uptake, Cheminformatics, Transporters

## Abstract

We bring together fifteen, nonredundant, tabulated collections (amounting to 696 separate measurements) of the apparent permeability (*P*_app_) of Caco-2 cells to marketed drugs. While in some cases there are some significant interlaboratory disparities, most are quite minor. Most drugs are not especially permeable through Caco-2 cells, with the median *P*_app_ value being some 16 ⋅ 10^−6^ cm s^−1^. This value is considerably lower than those (1,310 and 230 ⋅ 10^−6^ cm s^−1^) recently used in some simulations that purported to show that *P*_app_ values were too great to be transporter-mediated only. While these values are outliers, all values, and especially the comparatively low values normally observed, are entirely consistent with transporter-only mediated uptake, with no need to invoke phospholipid bilayer diffusion. The apparent permeability of Caco-2 cells to marketed drugs is poorly correlated with either simple biophysical properties, the extent of molecular similarity to endogenous metabolites (endogenites), or any specific substructural properties. In particular, the octanol:water partition coefficient, log*P*, shows negligible correlation with Caco-2 permeability. The data are best explained on the basis that most drugs enter (and exit) Caco-2 cells via a multiplicity of transporters of comparatively weak specificity.

## Introduction

Most pharmaceutical drugs, and all oral ones, must necessarily cross at least one cell membrane to act. Understanding how this transport is effected remains a major challenge (Kell & Oliver, 2014). We have brought together considerable published evidence (e.g., Dobson & Kell, 2008; Kell, 2013; Kell, 2015; Kell et al., 2013; Kell, Dobson & Oliver, 2011; Kell & Oliver, 2014) that suggests that (in contrast to the general textbook belief, e.g., Avdeef, 2012; Cao, Yu & Sun, 2006; Krogsgaard-Larsen, Liljefors & Madsen, 1996; Van De Waterbeemd & Testa, 2009) small molecule drugs ‘hitchhike’ on the many protein transporters (Kell, 2013; Kell & Goodacre, 2014; Sahoo et al., 2014; Thiele et al., 2013) that are part of normal intermediary metabolism. These transporters may be identified via experiments where gene expression levels are manipulated systematically as independent variables (César-Razquin et al., 2015; Giacomini et al., 2010; Han et al., 2015; Kell & Oliver, 2014; Lanthaler et al., 2011; Winter et al., 2014). A number of recent books summarise the importance of protein transport to drug disposition (Bhardwaj et al., 2008; Ecker & Chiba, 2009; Fromm & Kim, 2011; Ishikawa, Kim & König, 2013; Sugiyama & Steffansen, 2013; You & Morris, 2014).

Caco-2 cells (e.g., Artursson, Palm & Luthman, 2001; Awortwe, Fasinu & Rosenkranz, 2014; Balimane & Chong, 2005; Fearn & Hirst, 2006; Feng et al., 2014; Hidalgo, Raub & Borchardt, 1989; Sarmento et al., 2012; Sun et al., 2008; Van Breemen & Li, 2005; Volpe, 2011) are an epithelial cell line that has become a *de facto* standard in studies of pharmaceutical drug transport. They form a more or less (and otherwise) impermeable layer that is polarised, in the sense of having ‘apical’ and ‘basolateral’ faces in which transporters are differentially expressed. They express hundreds of transporters (Anderle, Huang & Sadée, 2004; Hayeshi et al., 2008; Landowski et al., 2004; Pshezhetsky et al., 2007; Sun et al., 2002), and (although far from perfect (Hilgendorf et al., 2007)) they have significant predictive power as to the fraction of oral dose absorbed in humans (e.g., Marino et al., 2005; Rubas et al., 1996).

It is thus of general interest to understand the kinds of apparent permeability (*P*_app_) rates for different drug molecules that Caco-2 cells can sustain. Although there are undoubtedly larger databases in-house in commercial and other enterprises, we have sought to bring together what we can of published data to determine the kinds of permeability values that Caco-2 cells can sustain, and what might determine that. We recognise that many factors can affect a specific measurement, e.g., the seeding density, age of the cells, pH and so on. An interlaboratory comparison (Hayeshi et al., 2008) indicated that while on occasion measurements could vary by more than an order of magnitude, overall the groupings were normally reasonably tight (say within a factor of 2–5).

The question of *P*_app_ values in Caco-2 cells has been brought into sharper focus by a recent article (Matsson et al., 2015a; Matsson et al., in press) that claimed unusually high rates for verapamil and propranolol, based on measurements in a specific earlier article (Avdeef et al., 2005) in which stirring had been performed at a massive rate (and one not used in any equivalent transporter kinetics measurements). We indicated that these values were major outliers (by one or even two orders of magnitude) (Mendes, Oliver & Kell, 2015), but did not pursue the question of what might be typical values of *P*_app_ for other drugs. This is the focus of what we do here.

## Methods

Data were extracted manually from tables in the papers stated, and compiled as an Excel sheet. Typical biophysical descriptors were added using the RDKit module (Riniker & Landrum, 2013) of KNIME (Berthold et al., 2008; Mazanetz et al., 2012; Saubern, Guha & Baell, 2011) (www.knime.org/), essentially as described (O’Hagan & Kell, 2015a; O’Hagan & Kell, 2015b; O’Hagan et al., 2015). For one experiment we used the CDK-KNIME nodes (Beisken et al., 2013).

**Figure 1:**
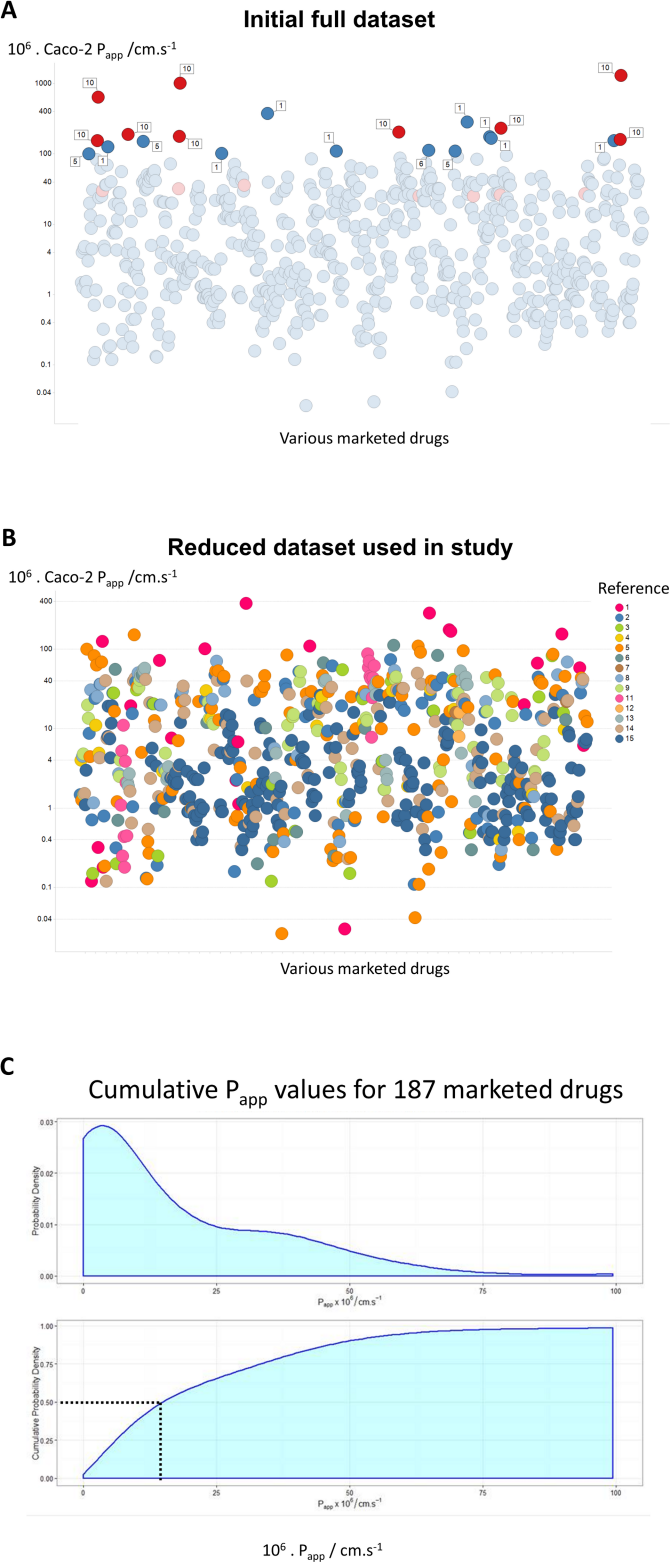
A compilation of 15 review articles on Caco-2 permeability measurements. (A) Full dataset, including outliers. (B) Reduced dataset after removal of the data from Avdeef et al. (2005). (C) Cumulative plot and smoothed histogram of the Caco-2 permeabilities in the reduced dataset. In (C) data for identical drugs were averaged. Data were extracted from the following papers. 1 (Bergström et al., 2003); 2 (Hou et al., 2004); 3 (Corti et al., 2006); 4 (Balimane, Han & Chong, 2006); 5 (Gozalbes et al., 2011); 6 (Peng et al., 2014); 7 (Press, 2011); 8 (Usansky & Sinko, 2005); 9 (Marino et al., 2005); 10 (Avdeef et al., 2005); 11 (Hayeshi et al., 2008); 12 (Wang et al., 2010); 13 (Uchida et al., 2009); 14 (Skolnik et al., 2010); 15 (Lin et al., 2011).

We have selected a set of 15 studies (indicated in the legend to Fig. 1) for our analysis. Based on the list of FDA-approved drugs that we downloaded (as before (O’Hagan & Kell, 2015b; O’Hagan et al., 2015)) from DrugBank (http://drugbank.ca) (Law et al., 2014), we compiled from these a non-redundant set of measurements of the apparent permeability (*P*_app_, that are commonly given in units of cm s^−1^). Although there are older papers, we have started with the compilation of Hou and colleagues (2004). Our method for avoiding redundancy in later compilations was not to include a separate measurement if the numbers given were identical to those in Hou et al. (2004) (or any other later papers) to at least 1 decimal place. We ignore any efflux transporters, since the evidence (that we show later) is that their influence on these measurements is fairly small (Lin et al., 2011). We incorporated two values from the review of Marino and colleagues (2005), one from lower throughput 24-well plates, one from a 96-well assay.

Where data were available for bidirectional assays, e.g., Hayeshi et al. (2008) and Skolnik et al. (2010), they are given just for the *A* → *B* direction. In the case of the interlaboratory comparison (Hayeshi et al., 2008), we used solely ‘batch 1’ data, while in the work of Lin et al. (2011), efflux inhibitors were sometimes present, as noted below. The entire dataset is given as an Excel sheet as a Table S1, and consists of 696 separate measurements. As indicated in Methods, we used KNIME to append some simple biophysical descriptors.

## Results

Figure 1A shows all of the data, with those studies finding rates above 100 ⋅ 10^−6^ cm s^−1^ labelled with the study number. Of the 21 measurements that have this property, no fewer than 9 (labelled in red) are from a study (Avdeef et al., 2005) of Avdeef and colleagues. The largest values (Avdeef et al., 2005) were observed at very high values of stirring rates (700 rpm), and these in particular contained a great many outliers. The implication is that these increases at exceptionally high stirring rates were due to unstirred layer effects, although it is hard to see their relevance to *in vivo* drug absorption where no such stirring is occurring. We also note (Dahlgren et al., 2015; Fagerholm & Lennernäs, 1995) that stirring has no effect on the transport of drugs through actual intestines. Mannitol is sometimes used as a membrane-impermeant control, taken to pass via a paracellular route. This said, mannitol controls did not always have the lowest values, and inulin (Marino et al., 2005) or EDTA (Lin et al., 2011) may be better. Although it was stated (Avdeef et al., 2005) that mannitol transport rates were ‘normal’, it is unclear why they do not change with stirring rates (or whether they do), so it is not entirely certain whether the epithelial layer remained intact, especially at some of the highest stirring rates employed. For these and other reasons, and especially given the strongly outlying nature of the measurements, we have decided for the rest of the analysis to exclude the data from Avdeef et al. (2005), resulting in an overall dataset of 680 separate measurements as shown in Fig. 1B. Although the *P*_app_ values might vary somewhat with the drug concentrations (e.g., Engman et al., 2003), we made no systematic attempt to take this into account, since (i) often the drug concentration values appearing in the Tables from which we took the data were not actually given, and (ii) this would not be expected to be by more than a factor 2, well within the range of variation seen in individual measurements. A cumulative plot and smoothed histogram of the data (Fig. 1C) shows that the most abundant values for *P*_app_ are in the range 3–4 ⋅ 10^−6^ cm s^−1^, and with a median value of ca 16 ⋅ 10^−6^ cm s^−1^. Obviously these values are considerably lower than those discussed in Matsson et al. (2015a) and Matsson et al. (in press), and indicate (Mendes, Oliver & Kell, 2015) that typical transporter kinetic parameters and expression levels are entirely adequate to account alone for cellular drug uptake, as proposed (Dobson et al., 2009; Dobson & Kell, 2008; Kell, 2013; Kell, 2015; Kell & Dobson, 2009; Kell et al., 2013; Kell, Dobson & Oliver, 2011; Kell & Goodacre, 2014; Kell & Oliver, 2014; Kell et al., 2015).

The chief point of this high-level, overview paper is that the values of *P*_app_ observed are typically rather low relative to those that can easily be explained on the basis of transporter-mediation only, without delving into minutiae. However, at the request of a reviewer we have added a Table (Table 1) that shows where available the concentrations of drug, insert type and stirring rates used in the relevant paper.

**Table 1:** Further details of the 15 transporter studies reviewed.

Drug concentration(s)	Insert type	Shaking or stirring speeds given	Reference
0.02–6 mM	Polycarbonate filter inserts, 12 mm diameter; pore size 0.4 μm; Costar	Mainly 500 rpm	Bergström et al. (2003)
Compilation of 13 references; not possible to deconstruct			Hou et al. (2004)
Not actually stated	Polycarbonate filters (area 1.13 cm^2^) in Costar Snapwell six-well plates	Not stated	Corti et al. (2006)
100–200 μM	Corning 24-well polycarbonate filter membrane (HTS-Transwell inserts, surface area: 0.33 cm^2^)	Not stated	Balimane, Han & Chong (2006)
10 μM	Fibrillar collagen coated PET membrane inserts in 24-well plates (BD Biosciences)	Not stated	Gozalbes et al. (2011)
10 μM	Collagen-coated 24-transwell plates	100 rpm	Peng et al. (2014)
1–10 μM	24-well systems from BD BioSciences (PET membrane, 1.0 mm, cat. #351181) or Costar (polycarbonate membrane, 0.4 mm, cat. #3396).	30 rpm	Press (2011)
Compilation; not possible to deconstruct			Usansky & Sinko (2005)
5 μM	‘Filter membranes’	Not stated	Marino et al. (2005)
50 nM–100 μM	Polycarbonate filter inserts (Transwell^®^ Costar; mean pore size 0.45 μm; diameters 12 mm)	25–7,000 rpm	Avdeef et al. (2005)
Mostly 30 μM, occasionally 100 μM	Polycarbonate, 0.4–3 μm, 6 mm or 12 mm	Not stated	Hayeshi et al. (2008)
10–500 μM	12-well Transwell plate with clear polyester membrane insert (0.4 μm pore diameter, 12 mm diameter), Corning Costar	50 rpm	Wang et al. (2010)
20 μM	‘Collagen-coated inserts’	Not stated	Uchida et al. (2009)
10 μM	“96-Multiwell Insert System from BD Biosciences”	Yes, but rate not stated	Skolnik et al. (2010)
10 μM	Six-well Transwell polycarbonate membrane inserts, Corning Life Science	Not stated	Lin et al. (2011)

**Figure 2:**
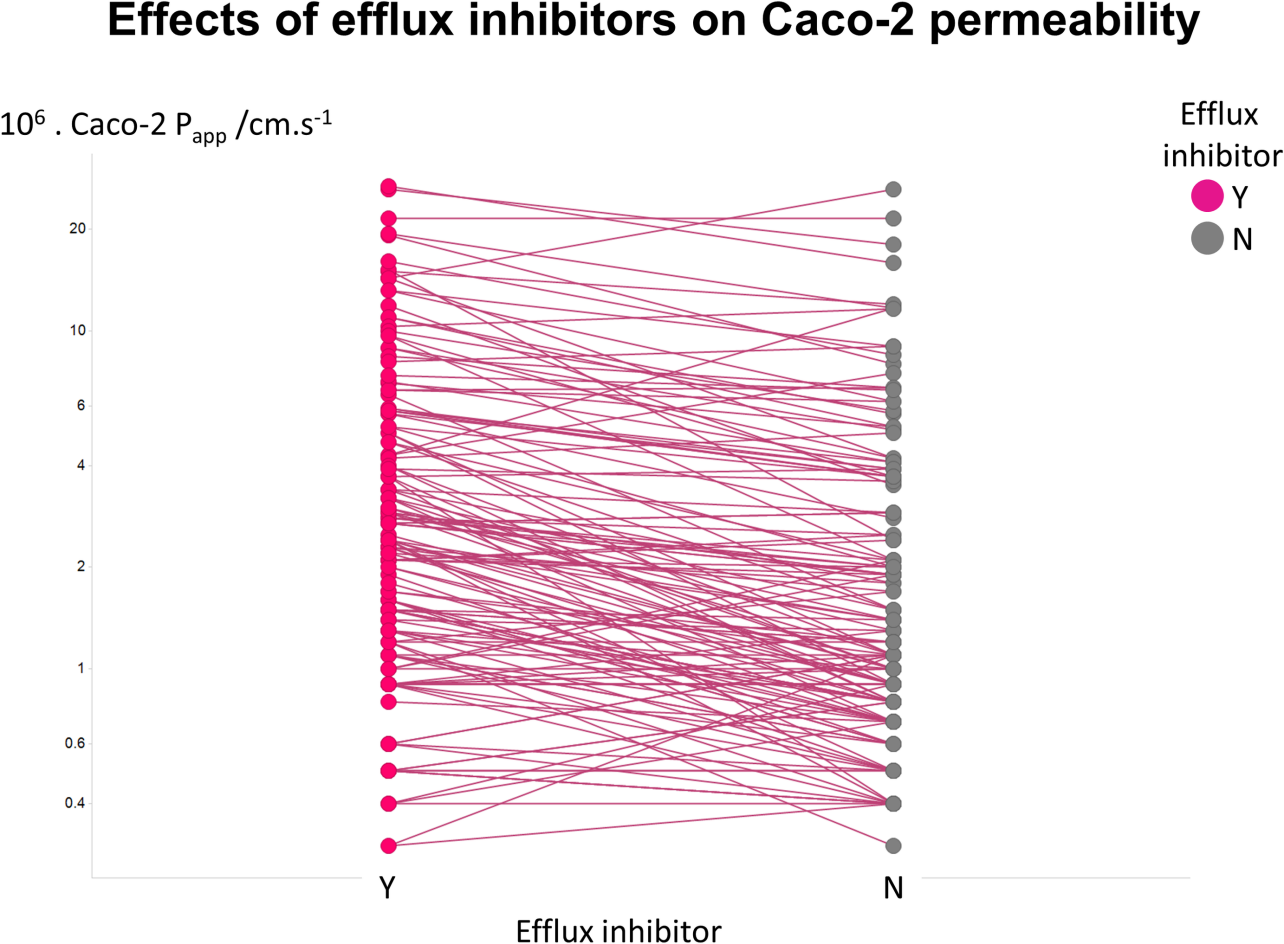
Relative lack of effect of efflux inhibitors on Caco-2 permeabilities of marketed drugs. Data are taken from Lin et al. (2011) and shown as paired values.

Figure 2 illustrates another feature of the data. Here we took the tabulated data of Lin and colleagues (2011) that used a variety of efflux inhibitors. A comparison showed that no very substantial (order-of-magnitude) differences in uptake were observed (Fig. 2), such that the typical ‘low’ values of *P*_app_ cannot realistically be ascribed to a major role of efflux pumps.

### Lack of relationship between Caco-2 permeability values and simple biophysical properties of drugs

**Figure 3:**
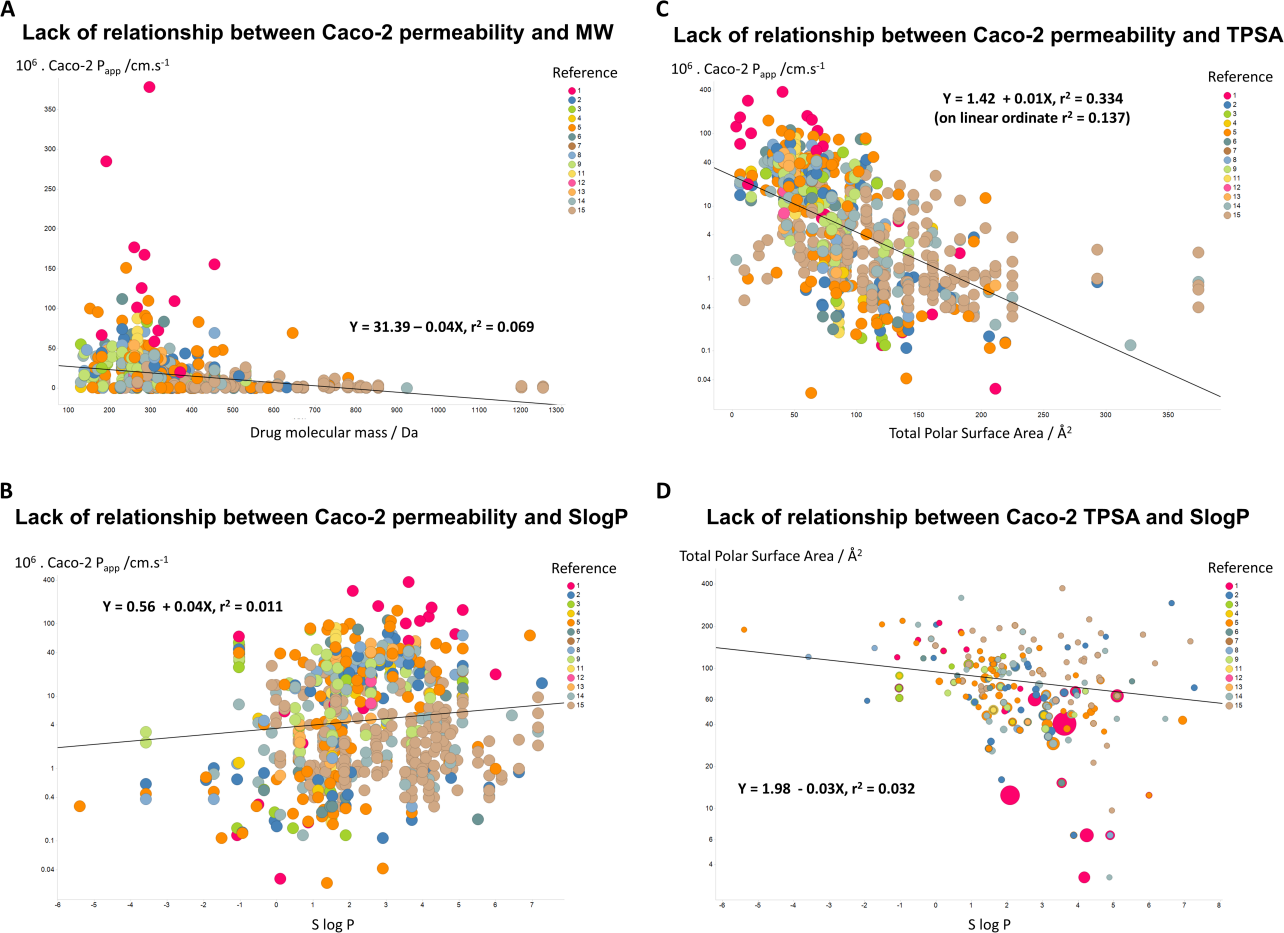
Lack of relationship between Caco-2 cells and simple biophysical parameters. (A) Caco-2 permeability as a function of MW. (B) Caco-2 permeability as a function of *S*log*P*. (C) Caco-2 permeability as a function of Total Polar Surface Area. (D) Lack of relationship between Total Polar Surface Area and Slog*P*. 5

If unstirred layer effects and pure diffusion (as opposed to transporter-based enzyme kinetics) were significant in Caco-2 permeability (notwithstanding the evidence that they are not (Fagerholm & Lennernäs, 1995)), one might suppose that permeability values should depend significantly upon the molecular mass of the drug involved. However, Fig. 3A shows that this is not the case, as the line of best fit has a slope of only −0.04X and a value for *r*^2^ of just 0.069. In a similar vein, despite a widespread view that transport rates should depend on log*P*, Fig. 3B shows that even when the Caco-2 permeabilities are plotted in log space, the *r*^2^ value for a plot against *S*log*P* is only 0.011. (For a plot in linear space the value drops to just *r*^2^ = 0.004, data not shown.) There is a slightly clearer relationship between Caco-2 permeability and a drug’s total polar surface area, but again the relationship is fairly weak (*r*^2^ = 0.334 when the ordinate is in log space, Fig. 3C, but only *r*^2^ = 0.137 when the ordinate is in linear space (plot not shown)). It is also of interest that there is no significant relationship between total Polar Surface Area and *S*log*P* (Fig. 3D). In particular, as before, we (e.g., Dobson & Kell, 2008; Kell & Oliver, 2014) and others (e.g., Skolnik et al., 2010) find that transmembrane permeability cannot be accounted for in terms of simple biophysical properties, and certainly not via log*P*.

### Lack of relationship between Caco-2 permeability and structural similarity to endogenous metabolites

**Figure 4:**
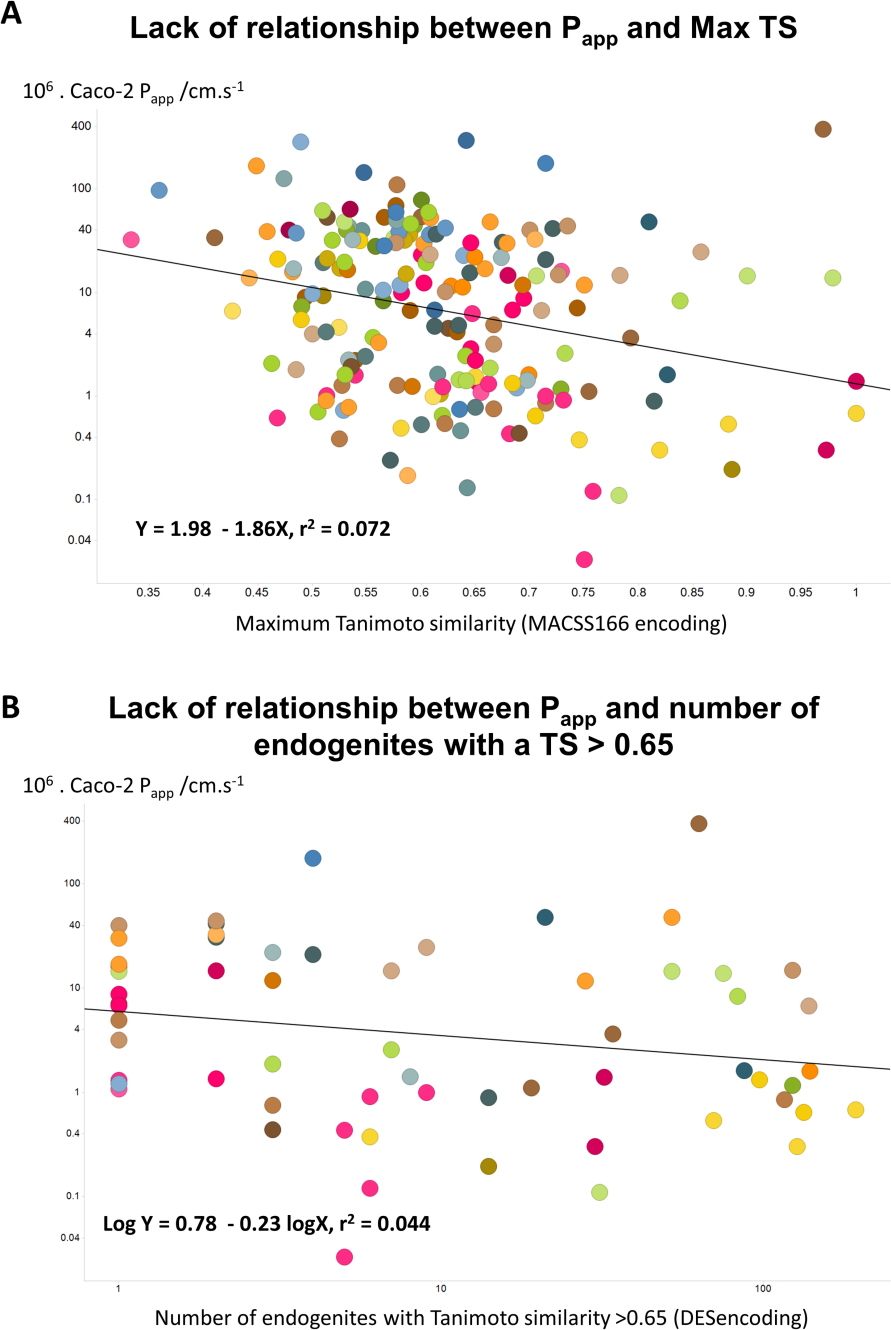
Lack of relationship between Caco-2 cell permeability and measures of endogenite-likeness. (A) Lack of relationship between the *P*_app_ of a drug in Caco-2 cells and its greatest Tanimoto similarity to any endogenite molecule in Recon2. (B) Lack of relationship between the *P*_app_ of a drug and the number of endogenous metabolites (endogenites) in Recon2 possessing a Tanimoto similarity greater than 0.65. 187 different drugs were assessed in each case.

Since the natural role of the transporters that drugs hitchhike on is to transport endogenous metaboliltes (Dobson & Kell, 2008; Kell, 2013; Kell, 2015; Kell et al., 2013; Kell & Oliver, 2014; Nigam, 2015; Swainston, Mendes & Kell, 2013), the ‘principle of molecular similarity’ (e.g., Bender & Glen, 2004; Eckert & Bajorath, 2007; Gasteiger, 2003; Maldonado et al., 2006) suggests that drugs should bear structural similarities to endogenous metabolites, and this is found to be the case (Dobson, Patel & Kell, 2009; O’Hagan & Kell, 2015b; O’Hagan et al., 2015). This led us to wonder whether any aspects of ‘metabolite-likeness’ might be related to Caco-2 permeability. However, we found no simple relationship of this type, whether (as illustrated) in terms of the closest Tanimoto similarity (Fig. 4A) or (for the 61 molecules for which this was true) the count of endogenites exceeding a Tanimoto similarity of 0.65 (Fig. 4B). (There was a very weak positive correlation, *r*^2^ = 0.156, with the number of endogenites exceeding a Tanimoto similarity of 0.75, for the 21 molecules that had at least one, data not shown.) One interpretation of this is that while in some cases a rather small number of transporters are typically involved in drug uptake (e.g., Winter et al., 2014), in many cases a considerably greater number contribute (e.g., Kell et al., 2013; Lanthaler et al., 2011). While well enough known in general (Mestres & Gregori-Puigjané, 2009), such ‘promiscuity’ has become much more manifest using modern chemical biology approaches to detect protein binding directly (e.g., Li et al., 2010; Niphakis et al., 2015).

**Figure 5:**
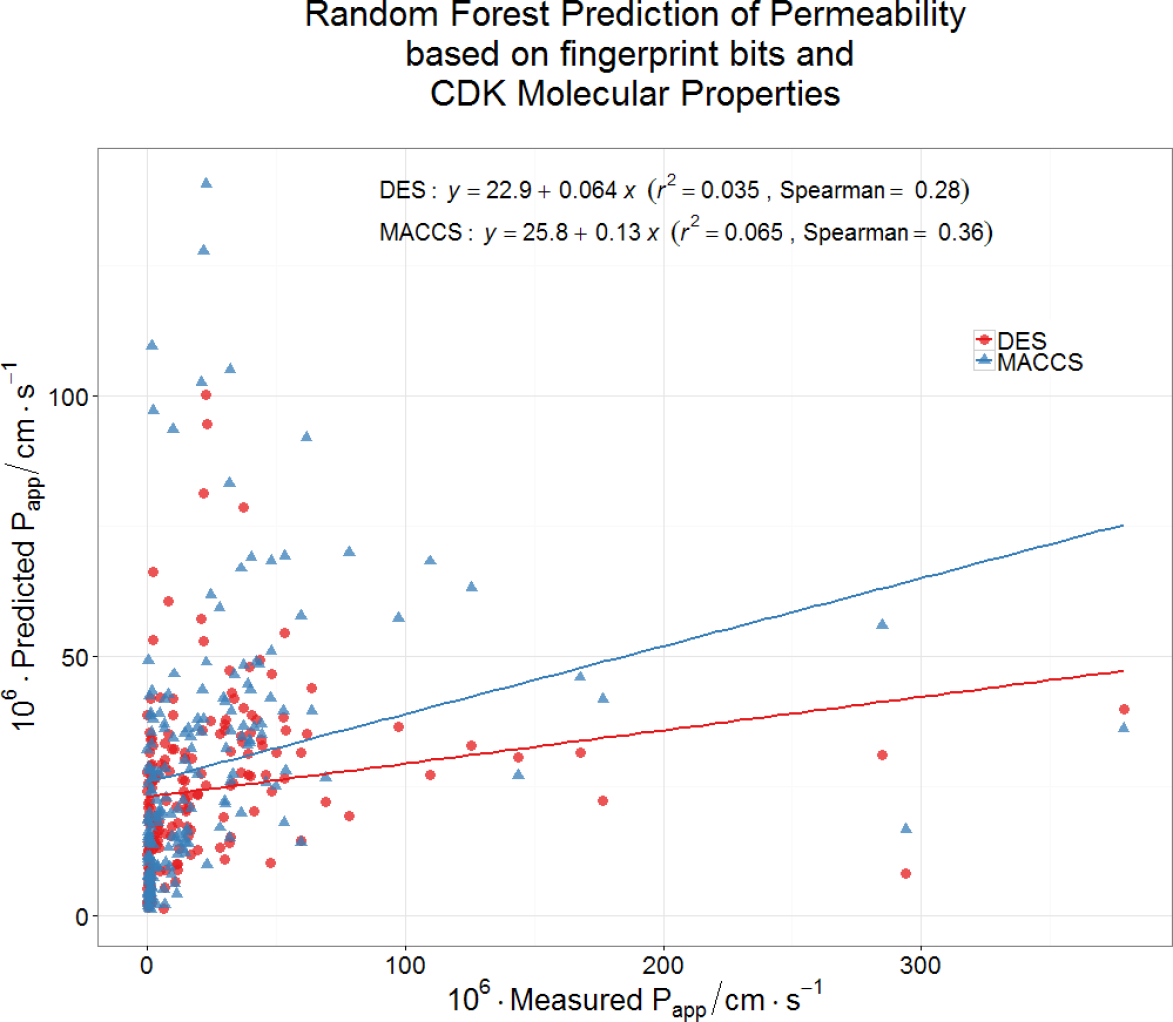
Lack of relationship between experimental Caco-2 permeabilities and those predicted (via out-of-bag estimation) from a random forest learner. Drug properties were encoded using either the MACCS166 encoding (O’Hagan et al., 2015) or the full DES encoding (O’Hagan & Kell, 2015b), each together with the molecular properties encoded in the CDK KNIME node (Beisken et al., 2013).

Finally, we wondered whether a standard machine learning approach (a random forest learner (Breiman, 2001; Fernández-Delgado et al., 2014; Knight et al., 2009; O’Hagan & Kell, 2015b)) might be able to predict Caco-2 permeabilities using a couple of fingerprint methods for encoding drug structures. Even this very powerful method had negligible predictive power as judged by its out-of-bag error (Fig. 5). It must be concluded that the ability to pass through Caco-2 cells is a very heterogeneous property, that cannot be accounted for via simple biophysical properties (e.g., those contributing to log*P*), and is best explained by the intermediacy of a very heterogeneous set of transporters.

## Discussion and Conclusions

A recent publication (Matsson et al., 2015a; Matsson et al., in press), using exceptionally high values of *P*_app_ for verapamil and propranolol, claimed that the apparent permeability values were such that they could not be supported by known (random) transporters at random expression level, *K_m_* and *k*_cat_ values. It was stated (Matsson et al., 2015a) that such rates “are possible in the absence of transmembrane diffusion, but only under very specific conditions that rarely or never occur for known human drug transporters”. While we showed that this was simply not the case (quite the opposite) (Mendes, Oliver & Kell, 2015), it prompted us to ask the question as to what typical rates of *P*_app_ might be for marketed drugs in Caco-2 cells more generally. By bringing together tabulated data from 15 studies, we found that the commonest values are just ca 3–4 ⋅ 10^−6^ cm s^−1^, and that the median value is ca 16 ⋅ 10^−6^ cm s^−1^. Thus, transporters alone can easily account for these. There was no significant correlation of *P*_app_ values with either the values of various biophysical descriptors or measures of endogenite-likeness, and even powerful machine learning methods could not predict the permeabilities from the drug structures. The most obvious reason for this is simply that there is no unitary explanation (such as simplistic phospholipid bilayer diffusion), as most drugs exploit multiple but often unknown transporters with overlapping specificities. Which they are and how much each contributes to a given Caco-2 permeability must be determined by varying their activities as independent variables (Kell, 2015; Kell & Oliver, 2014; Kell et al., 2015; César-Razquin et al., 2015), whether by using inhibitors (e.g., Han et al., 2015; Ming et al., 2009) or genetically. This latter activity has been initiated in other cell lines (e.g., Giacomini et al., 2010; Han et al., 2015; Lanthaler et al., 2011; Winter et al., 2014). The availability of powerful mammalian genome editing tools such as variants of the CRISPR/Cas9 system (e.g., Kleinstiver et al., 2015; Maeder et al., 2013; Wang et al., 2014; Zhou et al., 2014) imply that we may soon expect to see this strategy applied with great effect to the Caco-2 system.

## Supplemental Information

10.7717/peerj.1405/supp-1 Supplemental Information 1 Set of Caco-2 permeabilities and RDKit descriptors used herein Drugs_Caco2_compilation_with_descriptors_2.xls

## Additional Information and Declarations

### Data Availability

The following information was supplied regarding data availability: All the data we generated are provided in Supplemental Information 1.

## References

[ref-1] Anderle P, Huang Y, Sadée W (2004). Intestinal membrane transport of drugs and nutrients: genomics of membrane transporters using expression microarrays. European Journal of Pharmaceutical Science.

[ref-2] Artursson P, Palm K, Luthman K (2001). Caco-2 monolayers in experimental and theoretical predictions of drug transport. Advanced Drug Delivery Reviews.

[ref-3] Avdeef A (2012). Absorption and drug development: solubility, permeability and charge state.

[ref-4] Avdeef A, Artursson P, Neuhoff S, Lazorova L, Gråsjö J, Tavelin S (2005). Caco-2 permeability of weakly basic drugs predicted with the double-sink PAMPA pKa(flux) method. European Journal of Pharmaceutical Science.

[ref-5] Awortwe C, Fasinu PS, Rosenkranz B (2014). Application of Caco-2 cell line in herb-drug interaction studies: current approaches and challenges. Journal of Pharmacy & Pharmaceutical Sciences.

[ref-6] Balimane PV, Chong S (2005). Cell culture-based models for intestinal permeability: a critique. Drug Discovery Today.

[ref-7] Balimane PV, Han YH, Chong SH (2006). Current industrial practices of assessing permeability and P-glycoprotein interaction. The AAPS Journal.

[ref-8] Beisken S, Meinl T, Wiswedel B, De Figueiredo LF, Berthold M, Steinbeck C (2013). KNIME-CDK: workflow-driven cheminformatics. BMC Bioinformatics.

[ref-9] Bender A, Glen RC (2004). Molecular similarity: a key technique in molecular informatics. Organic and Biomolecular Chemistry.

[ref-10] Bergström CAS, Strafford M, Lazorova L, Avdeef A, Luthman K, Artursson P (2003). Absorption classification of oral drugs based on molecular surface properties. Journal of Medicinal Chemistry.

[ref-11] Berthold MR, Cebron N, Dill F, Gabriel TR, Kötter T, Meinl T, Ohl P, Sieb C, Thiel K, Wiswedel B, Preisach C, Burkhardt H, Schmidt-Thieme L, Decker R (2008). KNIME: the Konstanz information miner. Data analysis, machine learning and applications.

[ref-12] Bhardwaj RK, Herrera-Ruiz DR, Xu Y, Carl SM, Cook TJ, Vorsa N, Knipp GT, Krishna R, Yu L (2008). Intestinal transporters in drug absorption. Biopharmaceutics applications in drug development.

[ref-13] Breiman L (2001). Random forests. Machine Learning.

[ref-14] Cao X, Yu LX, Sun D, Krishna R, Yu L (2006). Drug absorption principles. Biopharmaceutics applications in drug development.

[ref-15] César-Razquin A, Snijder B, Frappier-Brinton T, Isserlin R, Gyimesi G, Bai X, Reithmeier RA, Hepworth D, Hediger MA, Edwards AM, Superti-Furga G (2015). A call for systematic research on solute carriers. Cell.

[ref-16] Corti G, Maestrelli F, Cirri M, Zerrouk N, Mura P (2006). Development and evaluation of an *in vitro* method for prediction of human drug absorption—II. Demonstration of the method suitability. European Journal of Pharmaceutical Science.

[ref-17] Dahlgren D, Roos C, Sjögren E, Lennernäs H (2015). Direct *in vivo* human intestinal permeability (*p*_eff_) determined with different clinical perfusion and intubation methods. Journal of Pharmaceutical Sciences.

[ref-18] Dobson PD, Kell DB (2008). Carrier-mediated cellular uptake of pharmaceutical drugs: an exception or the rule?. Nature Reviews Drug Discovery.

[ref-19] Dobson P, Lanthaler K, Oliver SG, Kell DB (2009). Implications of the dominant role of cellular transporters in drug uptake. Current Topics in Medicinal Chemistry.

[ref-20] Dobson PD, Patel Y, Kell DB (2009). “Metabolite-likeness” as a criterion in the design and selection of pharmaceutical drug libraries. Drug Discovery Today.

[ref-21] Ecker G, Chiba P (2009). Transporters as drug carriers: structure function, substrates.

[ref-22] Eckert H, Bajorath J (2007). Molecular similarity analysis in virtual screening: foundations, limitations and novel approaches. Drug Discovery Today.

[ref-23] Engman H, Tannergren C, Artursson P, Lennernäs H (2003). Enantioselective transport and CYP3A4-mediated metabolism of R/S-verapamil in Caco-2 cell monolayers. European Journal of Pharmaceutical Science.

[ref-24] Fagerholm U, Lennernäs H (1995). Experimental estimation of the effective unstirred water layer thickness in the human jejunum, and its importance in oral drug absorption. European Journal of Pharmaceutical Science.

[ref-25] Fearn RA, Hirst BH (2006). Predicting oral drug absorption and hepatobiliary clearance: human intestinal and hepatic *in vitro* cell models. Environmental Toxicology and Pharmacology.

[ref-26] Feng B, Varma MV, Costales C, Zhang H, Tremaine L (2014). *In vitro* and *in vivo* approaches to characterize transporter-mediated disposition in drug discovery. Expert Opinion on Drug Discovery.

[ref-27] Fernández-Delgado M, Cernadas E, Barro S, Amorim D (2014). Do we need hundreds of classifiers to solve real world classification problems?. Journal of Machine Learning Research.

[ref-28] Fromm MF, Kim RB (2011). Drug transporters. Handbook of experimental pharmacology.

[ref-29] Gasteiger J (2003). Handbook of chemoinformatics: from data to knowledge.

[ref-30] Giacomini KM, Huang SM, Tweedie DJ, Benet LZ, Brouwer KL, Chu X, Dahlin A, Evers R, Fischer V, Hillgren KM, Hoffmaster KA, Ishikawa T, Keppler D, Kim RB, Lee CA, Niemi M, Polli JW, Sugiyama Y, Swaan PW, Ware JA, Wright SH, Wah Yee S, Zamek-Gliszczynski MJ, Zhang L (2010). Membrane transporters in drug development. Nature Reviews Drug Discovery.

[ref-31] Gozalbes R, Jacewicz M, Annand R, Tsaioun K, Pineda-Lucena A (2011). QSAR-based permeability model for drug-like compounds. Bioorganic and Medicinal Chemistry.

[ref-32] Han TK, Proctor WR, Costales CL, Cai H, Everett RS, Thakker DR (2015). Four cation-selective transporters contribute to apical uptake and accumulation of metformin in Caco-2 cell monolayers. Journal of Pharmacology and Experimental Therapeutics.

[ref-33] Hayeshi R, Hilgendorf C, Artursson P, Augustijns P, Brodin B, Dehertogh P, Fisher K, Fossati L, Hovenkamp E, Korjamo T, Masungi C, Maubon N, Mols R, Müllertz A, Mönkkönen J, O’Driscoll C, Oppers-Tiemissen HM, Ragnarsson EG, Rooseboom M, Ungell AL (2008). Comparison of drug transporter gene expression and functionality in Caco-2 cells from 10 different laboratories. European Journal of Pharmaceutical Science.

[ref-34] Hidalgo IJ, Raub TJ, Borchardt RT (1989). Characterization of the human colon carcinoma cell line (Caco-2) as a model system for intestinal epithelial permeability. Gastroenterology.

[ref-35] Hilgendorf C, Ahlin G, Seithel A, Artursson P, Ungell AL, Karlsson J (2007). Expression of thirty-six drug transporter genes in human intestine, liver, kidney, and organotypic cell lines. Drug Metabolism and Disposition.

[ref-36] Hou TJ, Zhang W, Xia K, Qiao XB, Xu XJ (2004). ADME evaluation in drug discovery. 5. Correlation of Caco-2 permeation with simple molecular properties. Journal of Chemical Information and Computer Sciences.

[ref-37] Ishikawa T, Kim RB, König J (2013). Pharmacogenomics of human drug transporters: clinical impacts.

[ref-38] Kell DB (2013). Finding novel pharmaceuticals in the systems biology era using multiple effective drug targets, phenotypic screening, and knowledge of transporters: where drug discovery went wrong and how to fix it. The FEBS Journal.

[ref-39] Kell DB (2015). What would be the observable consequences if phospholipid bilayer diffusion of drugs into cells is negligible?. Trends in Pharmacological Sciences.

[ref-40] Kell DB, Dobson PD, Hicks MG, Kettner C (2009). The cellular uptake of pharmaceutical drugs is mainly carrier-mediated and is thus an issue not so much of biophysics but of systems biology. Proc int beilstein symposium on systems chemistry.

[ref-41] Kell DB, Dobson PD, Bilsland E, Oliver SG (2013). The promiscuous binding of pharmaceutical drugs and their transporter-mediated uptake into cells: what we (need to) know and how we can do so. Drug Discovery Today.

[ref-42] Kell DB, Dobson PD, Oliver SG (2011). Pharmaceutical drug transport: the issues and the implications that it is essentially carrier-mediated only. Drug Discovery Today.

[ref-43] Kell DB, Goodacre R (2014). Metabolomics and systems pharmacology: why and how to model the human metabolic network for drug discovery. Drug Discovery Today.

[ref-44] Kell DB, Oliver SG (2014). How drugs get into cells: tested and testable predictions to help discriminate between transporter-mediated uptake and lipoidal bilayer diffusion. Frontiers in Pharmacology.

[ref-45] Kell DB, Swainston N, Pir P, Oliver SG (2015). Membrane transporter engineering in industrial biotechnology and whole-cell biocatalysis. Trends in Biotechnology.

[ref-46] Kleinstiver BP, Prew MS, Tsai SQ, Topkar VV, Nguyen NT, Zheng Z, Gonzales APW, Li Z, Peterson RT, Yeh JR, Aryee MJ, Joung JK (2015). Engineered CRISPR-Cas9 nucleases with altered PAM specificities. Nature.

[ref-47] Knight CG, Platt M, Rowe W, Wedge DC, Khan F, Day P, McShea A, Knowles J, Kell DB (2009). Array-based evolution of DNA aptamers allows modelling of an explicit sequence-fitness landscape. Nucleic Acids Research.

[ref-48] Krogsgaard-Larsen P, Liljefors T, Madsen U (1996). A textbook of drug design and development.

[ref-49] Landowski CP, Anderle P, Sun D, Sadee W, Amidon GL (2004). Transporter and ion channel gene expression after Caco-2 cell differentiation using 2 different microarray technologies. The AAPS Journal.

[ref-50] Lanthaler K, Bilsland E, Dobson P, Moss HJ, Pir P, Kell DB, Oliver SG (2011). Genome-wide assessment of the carriers involved in the cellular uptake of drugs: a model system in yeast. BMC Biology.

[ref-51] Law V, Knox C, Djoumbou Y, Jewison T, Guo AC, Liu Y, Maciejewski A, Arndt D, Wilson M, Neveu V, Tang A, Gabriel G, Ly C, Adamjee S, Dame ZT, Han B, Zhou Y, Wishart DS (2014). DrugBank 4.0: shedding new light on drug metabolism. Nucleic Acids Research.

[ref-52] Li X, Gianoulis TA, Yip KY, Gerstein M, Snyder M (2010). Extensive *in vivo* metabolite–protein interactions revealed by large-scale systematic analyses. Cell.

[ref-53] Lin X, Skolnik S, Chen X, Wang J (2011). Attenuation of intestinal absorption by major efflux transporters: quantitative tools and strategies using a Caco-2 model. Drug Metabolism and Disposition.

[ref-54] Maeder ML, Linder SJ, Cascio VM, Fu Y, Ho QH, Joung JK (2013). CRISPR RNA-guided activation of endogenous human genes. Nature Methods.

[ref-55] Maldonado AG, Doucet JP, Petitjean M, Fan BT (2006). Molecular similarity and diversity in chemoinformatics: from theory to applications. Molecular Divers.

[ref-56] Marino AM, Yarde M, Patel H, Chong S, Balimane PV (2005). Validation of the 96 well Caco-2 cell culture model for high throughput permeability assessment of discovery compounds. International Journal of Phamaceutics.

[ref-57] Matsson P, Fenu LA, Lundquist P, Wisńiewski JR, Kansy M, Artursson P (2015a). Quantifying the impact of transporters on cellular drug permeability. Trends in Pharmacological Sciences.

[ref-58] Matsson P, Fenu LA, Lundquist P, Wisńiewski JR, Kansy M, Artursson P (2015). Supplementary Information: addendum to ‘Quantifying the impact of transporters on cellular drug permeability’. Trends in Pharmacological Sciences.

[ref-59] Mazanetz MP, Marmon RJ, Reisser CBT, Morao I (2012). Drug discovery applications for KNIME: an open source data mining platform. Current Topics in Medicinal Chemistry.

[ref-60] Mendes P, Oliver SG, Kell DB (2015). Fitting transporter activities to cellular drug concentrations and fluxes: why the bumblebee can fly. Trends in Pharmacological Sciences.

[ref-61] Mestres J, Gregori-Puigjané E (2009). Conciliating binding efficiency and polypharmacology. Trends in Pharmacological Sciences.

[ref-62] Ming X, Ju W, Wu H, Tidwell RR, Hall JE, Thakker DR (2009). Transport of dicationic drugs pentamidine and furamidine by human organic cation transporters. Drug Metabolism and Disposition.

[ref-63] Nigam SK (2015). What do drug transporters really do?. Nature Reviews Drug Discovery.

[ref-64] Niphakis MJ, Lum KM, Cognetta AB, Correia BE, Ichu TA, Olucha J, Brown SJ, Kundu S, Piscitelli F, Rosen H, Cravatt BF (2015). A global map of lipid-binding proteins and their ligandability in cells. Cell.

[ref-65] O’Hagan S, Kell DB (2015a). Software review: the KNIME workflow environment and its applications in Genetic Programming and machine learning. Genetic Programming and Evolvable Machines.

[ref-66] O’Hagan S, Kell DB (2015b). Understanding the foundations of the structural similarities between marketed drugs and endogenous human metabolites. Frontiers in Pharmacology.

[ref-67] O’Hagan S, Swainston N, Handl J, Kell DB (2015). A ‘rule of 0.5’ for the metabolite-likeness of approved pharmaceutical drugs. Metabolomics.

[ref-68] Peng Y, Yadava P, Heikkinen AT, Parrott N, Railkar A (2014). Applications of a 7-day Caco-2 cell model in drug discovery and development. European Journal of Pharmaceutical Science.

[ref-69] Press B (2011). Optimization of the Caco-2 permeability assay to screen drug compounds for intestinal absorption and efflux. Methods in Molecular Biology.

[ref-70] Pshezhetsky AV, Fedjaev M, Ashmarina L, Mazur A, Budman L, Sinnett D, Labuda D, Beaulieu JF, Menard D, Nifant’ev I, Levy E (2007). Subcellular proteomics of cell differentiation: quantitative analysis of the plasma membrane proteome of Caco-2 cells. Proteomics.

[ref-71] Riniker S, Landrum GA (2013). Open-source platform to benchmark fingerprints for ligand-based virtual screening. Journal of Cheminformatics.

[ref-72] Rubas W, Cromwell ME, Shahrokh Z, Villagran J, Nguyen TN, Wellton M, Nguyen TH, Mrsny RJ (1996). Flux measurements across Caco-2 monolayers may predict transport in human large intestinal tissue. Journal of Pharmaceutical Sciences.

[ref-73] Sahoo S, Aurich MK, Jonsson JJ, Thiele I (2014). Membrane transporters in a human genome-scale metabolic knowledgebase and their implications for disease. Frontiers in Physiology.

[ref-74] Sarmento B, Andrade F, Da Silva SB, Rodrigues F, Das Neves J, Ferreira D (2012). Cell-based *in vitro* models for predicting drug permeability. Expert Opinion on Drug Metabolism & Toxicology.

[ref-75] Saubern S, Guha R, Baell JB (2011). KNIME workflow to assess PAINS filters in SMARTS Format. Comparison of RDKit and indigo cheminformatics libraries. Molecular Informatics.

[ref-76] Skolnik S, Lin X, Wang J, Chen XH, He T, Zhang B (2010). Towards prediction of *in vivo* intestinal absorption using a 96-well Caco-2 assay. Journal of Pharmaceutical Sciences.

[ref-77] Sugiyama Y, Steffansen B (2013). Transporters in drug development: discovery, optimization, clinical study and regulation.

[ref-78] Sun H, Chow EC, Liu S, Du Y, Pang KS (2008). The Caco-2 cell monolayer: usefulness and limitations. Expert Opinion on Drug Metabolism & Toxicology.

[ref-79] Sun D, Lennernäs H, Welage LS, Barnett JL, Landowski CP, Foster D, Fleisher D, Lee KD, Amidon GL (2002). Comparison of human duodenum and Caco-2 gene expression profiles for 12,000 gene sequences tags and correlation with permeability of 26 drugs. Pharmaceutical Research.

[ref-80] Swainston N, Mendes P, Kell DB (2013). An analysis of a ‘community-driven’ reconstruction of the human metabolic network. Metabolomics.

[ref-81] Thiele I, Swainston N, Fleming RMT, Hoppe A, Sahoo S, Aurich MK, Haraldsdottír H, Mo ML, Rolfsson O, Stobbe MD, Thorleifsson SG, Agren R, Bölling C, Bordel S, Chavali AK, Dobson P, Dunn WB, Endler L, Goryanin I, Hala D, Hucka M, Hull D, Jameson D, Jamshidi N, Jones J, Jonsson JJ, Juty N, Keating S, Nookaew I, Le Novère N, Malys N, Mazein A, Papin JA, Patel Y, Price ND, Selkov E, Sigurdsson MI, Simeonidis E, Sonnenschein N, Smallbone K, Sorokin A, Beek HV, Weichart D, Nielsen JB, Westerhoff HV, Kell DB, Mendes P, Palsson BØ (2013). A community-driven global reconstruction of human metabolism. Nature Biotechnology.

[ref-82] Uchida M, Fukazawa T, Yamazaki Y, Hashimoto H, Miyamoto Y (2009). A modified fast (4 day) 96-well plate Caco-2 permeability assay. Journal of Pharmacological Toxicological Methods.

[ref-83] Usansky HH, Sinko PJ (2005). Estimating human drug oral absorption kinetics from Caco-2 permeability using an absorption-disposition model: model development and evaluation and derivation of analytical solutions for k_*a*_ and F_*a*_. Journal of Pharmacology and Experimental Therapeutics.

[ref-84] Van Breemen RB, Li Y (2005). Caco-2 cell permeability assays to measure drug absorption. Expert Opinion on Drug Metabolism & Toxicology.

[ref-85] Van De Waterbeemd H, Testa B (2009). Drug bioavailability: estimation of solubility, permeability, absorption and bioavailability.

[ref-86] Volpe DA (2011). Drug-permeability and transporter assays in Caco-2 and MDCK cell lines. Future Medicinal Chemistry.

[ref-87] Wang Y, Cao J, Wang X, Zeng S (2010). Stereoselective transport and uptake of propranolol across human intestinal Caco-2 cell monolayers. Chirality.

[ref-88] Wang T, Wei JJ, Sabatini DM, Lander ES (2014). Genetic screens in human cells using the CRISPR-Cas9 system. Science.

[ref-89] Winter GE, Radic B, Mayor-Ruiz C, Blomen VA, Trefzer C, Kandasamy RK, Huber KVM, Gridling M, Chen D, Klampfl T, Kralovics R, Kubicek S, Fernandez-Capetillo O, Brummelkamp TR, Superti-Furga G (2014). The solute carrier SLC35F2 enables YM155-mediated DNA damage toxicity. Nature Chemical Biology.

[ref-90] You G, Morris ME, Wang B (2014). Drug transporters: molecular characterization and role in drug disposition.

[ref-91] Zhou Y, Zhu S, Cai C, Yuan P, Li C, Huang Y, Wei W (2014). High-throughput screening of a CRISPR/Cas9 library for functional genomics in human cells. Nature.

